# Primary pulmonary monophasic synovial sarcoma initially presenting with bloody pleural effusion: A case report and literature review

**DOI:** 10.1002/ccr3.8841

**Published:** 2024-04-26

**Authors:** Tengcheng Yin, Bing Liu, Jinru Xue, Xiyu Liu, Shengtao Shang, Yan Wang

**Affiliations:** ^1^ Thoracic Surgery, China‐Japan Union Hospital of Jilin University Changchun China; ^2^ Emergency, The Third Affiliated Hospital of Changchun University of Chinese Medicine Changchun China

**Keywords:** bloody pleural effusion, case report, lung tumors, monophasic synovial sarcoma

## Abstract

**Key Clinical Message:**

Primary pulmonary synovial sarcoma (PPSS) can originate from blood vessels of the bronchial wall, lung interstitium, and interstitial components, and accounts for 0.1%–0.5% of all primary lung malignancies, the most common symptoms are chest pain, cough, dyspnea, and hemoptysis.

**Abstract:**

Synovial sarcoma (SS) is a rare malignant tumor of stromal origin, which accounts for approximately 8%–10% of all soft tissue sarcomas. Primary pulmonary synovial sarcoma (PPSS) can originate from blood vessels of the bronchial wall, lung interstitium, and interstitial components, and accounts for 0.1%–0.5% of all primary lung malignancies. Patient concerns: We report the first case of a 57‐year‐old man with bloody pleural effusion as an initial manifestation of PPSS in the middle lobe of the right lung diagnosed after surgery. Diagnosis: Chest computed tomography (CT) revealed a mass in the middle lobe of the right lung, which was pathologically diagnosed as a monophasic SS after surgical resection. Interventions: Ten days after preoperative closed chest drainage, a right thoracotomy was performed to remove the right middle lobe of the lung. Outcomes: The patient recovered smoothly and was discharged from the hospital without any other postoperative treatment. A follow‐up chest CT scan 7 months postoperatively revealed intrapulmonary recurrence with multiple metastases. Lessons: Monophasic PPSS of the lung may present with bloody pleural effusion as its first manifestation.

## Graphical abstract

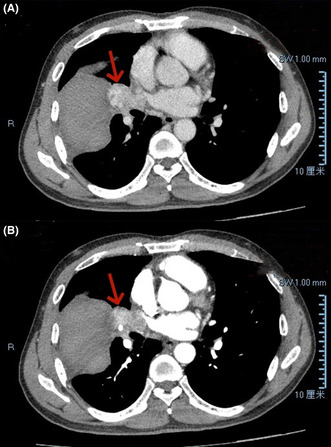

## 1 INTRODUCTION

Synovial sarcoma (SS) is a rare mesenchymal malignant tumor, accounting for approximately 5%–10% of soft tissue sarcomas,^1^ and can occur in any anatomical location. Primary intrathoracic SS is very rare, accounting for 0.1%–0.5% of all lung tumors.^2^ A significant genetic feature of SS is the translocation of the causative factor t(X;18) (p11;Q11), resulting in fusion of the SS18 gene (SYT) on chromosome 18 to one of the three highly homologous SSX genes (SSX1, SSX2, or SSX4), all of which are on the X chromosome.^3^ SSs are classified into pure sarcomas and biphasic SSs (a combination of epithelioid and sarcomatous components).^3^

We report the case of a 57‐year‐old man who presented with right bloody pleural effusion and a right middle lobe mass as the initial manifestation. Postoperative pathology confirmed monophasic SS. In addition, we provide a review of the clinical manifestations, diagnosis, and treatment of this disorder using the relevant literature.

## 2 CASE PRESENTATION

**FIGURE 1:**
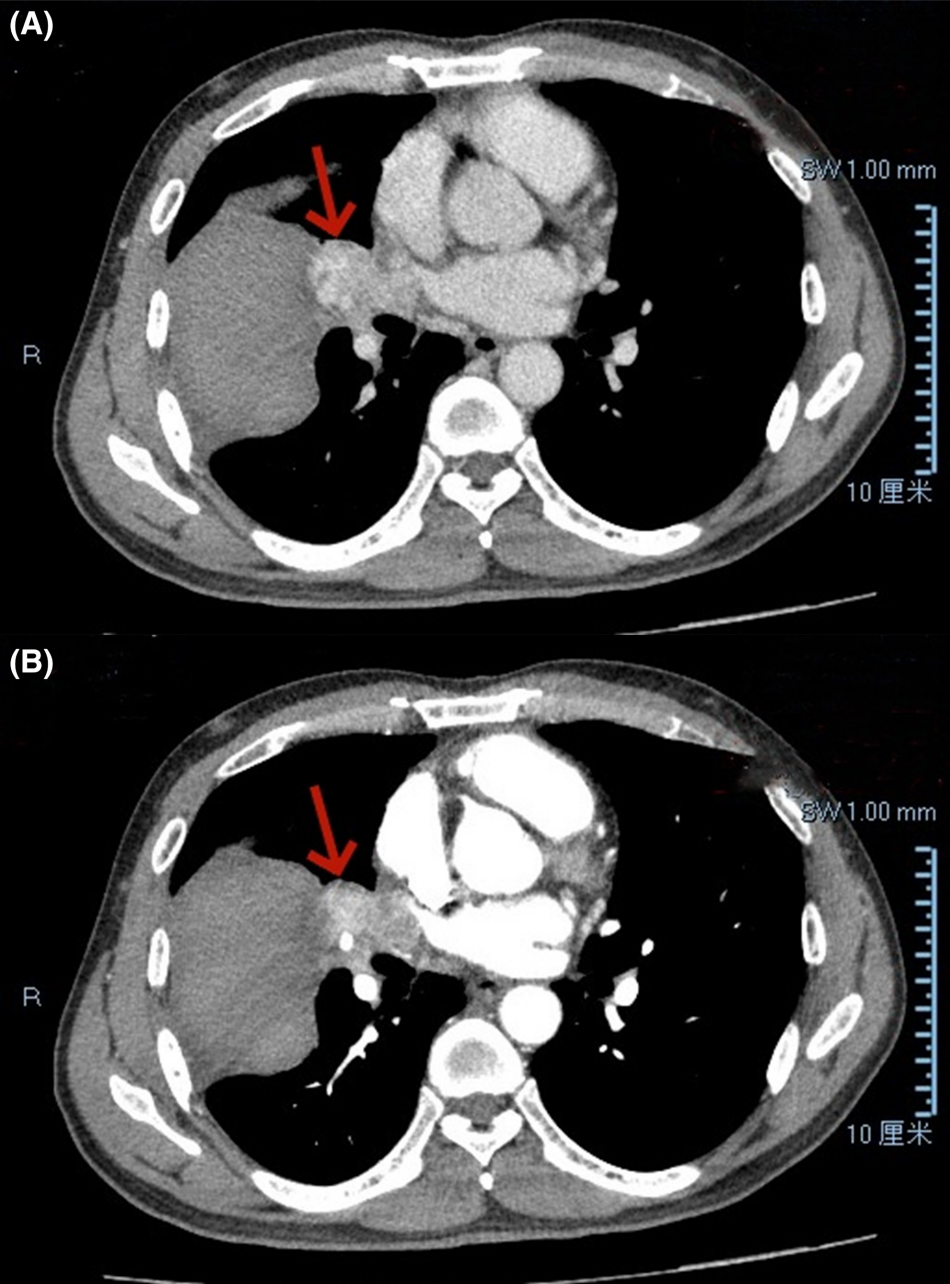
The chest contrast‐enhanced computed tomography (CT) image showed a mass in the right middle lobe of the lung near the hilus and a right horizontal interlobar hematoma. (A) Shows the venous phase appearance of chest enhanced CT; (B) Shows the arterial phase appearance.

A 57‐year‐old man was admitted to our hospital with dyspnea for 15 days, hemoptysis, and syncope twice, with no family history. He did not receive treatment before coming to our hospital. Chest computed tomography (CT) performed in the outpatient department of our hospital the day before admission showed a mass shadow next to the middle lobe of the right lung, approximately 2.3 cm × 2.9 cm in size, and the enhanced scan showed delayed enhancement (Figure 1). The possibility of neoplastic lesions was considered high, and vascular tumors were not excluded. There was a patchy abnormal enhancement in the horizontal fissure of the right lung with clear edges and a size of approximately 8.3 cm × 5.6 cm × 4.1 cm, which was considered to be a hematoma. Under ultrasound guidance, a thoracentesis catheter was inserted to drain approximately 1000 mL of bleeding fluid.

On admission, the patients' blood pressure was 145/84 mmHg, heart rate was 78 beats/min, breathing was 16 breaths/min, and body temperature was 36.5°C. No cancer cells were found in the exfoliated cells of the pleural effusion. No obvious abnormalities were found during bronchoscopy and no abnormalities were found during other systemic examinations.

### 2.1 Treatment

**FIGURE 2:**
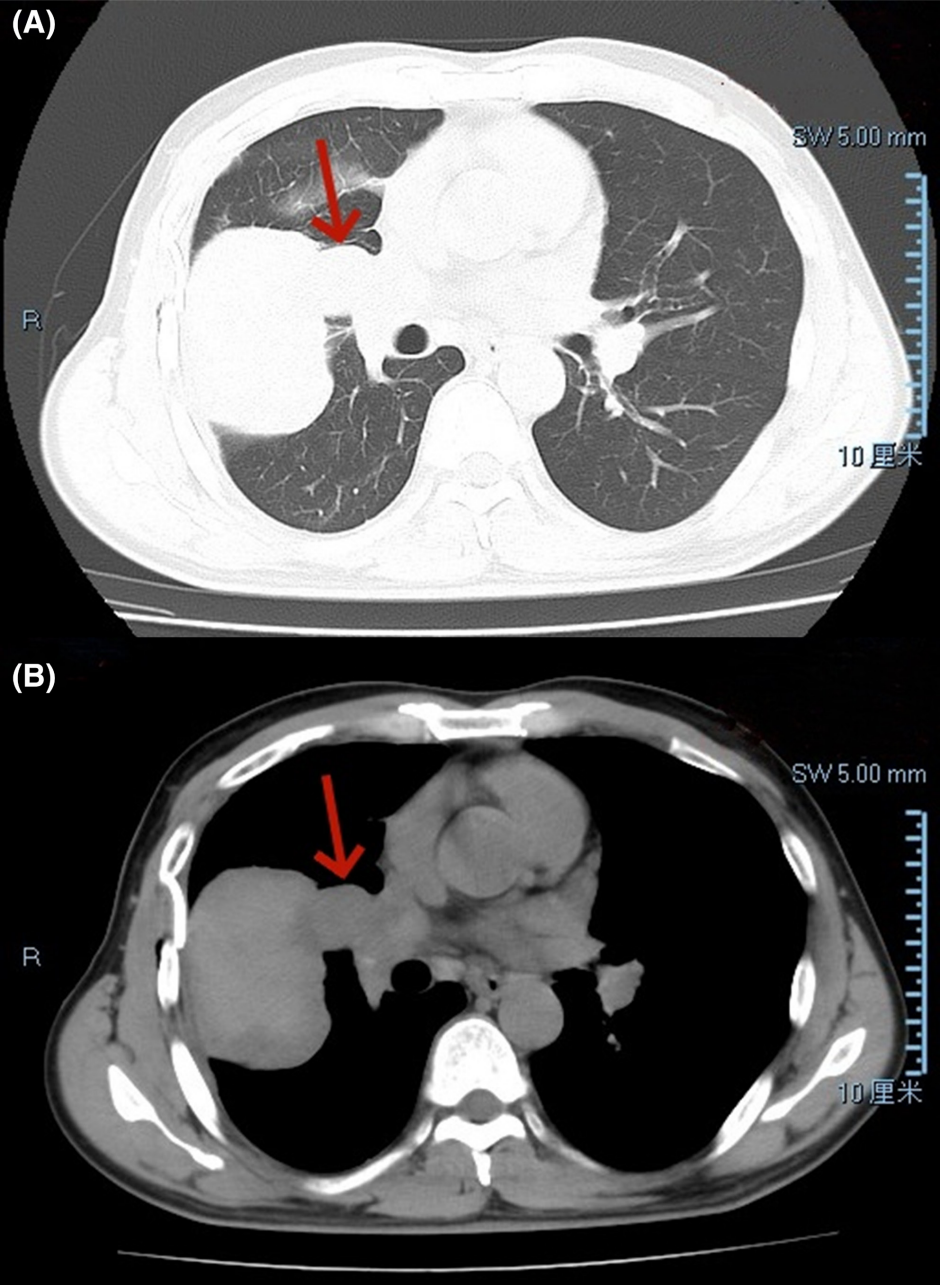
CT after drainage showed no significant change in the mass. (A) Shows the lung window manifestation on chest CT plain scan, and (B) Shows the mediastinal window manifestation.

Chest CT reexamination 7 days later showed increased pleural effusion on the right side, but no change in the abovementioned mass (Figure 2). During surgery, the patient was placed in the left lateral decubitus position and underwent double‐lumen endotracheal intubation. A fifth intercostal incision on the right side was used as the operating hole for chest exploration. There was a bloody effusion in the pleural cavity and a tough mass in the middle lobe of the right lung. After complete removal of the mass, old intrapulmonary blood clots were observed.

**FIGURE 3:**
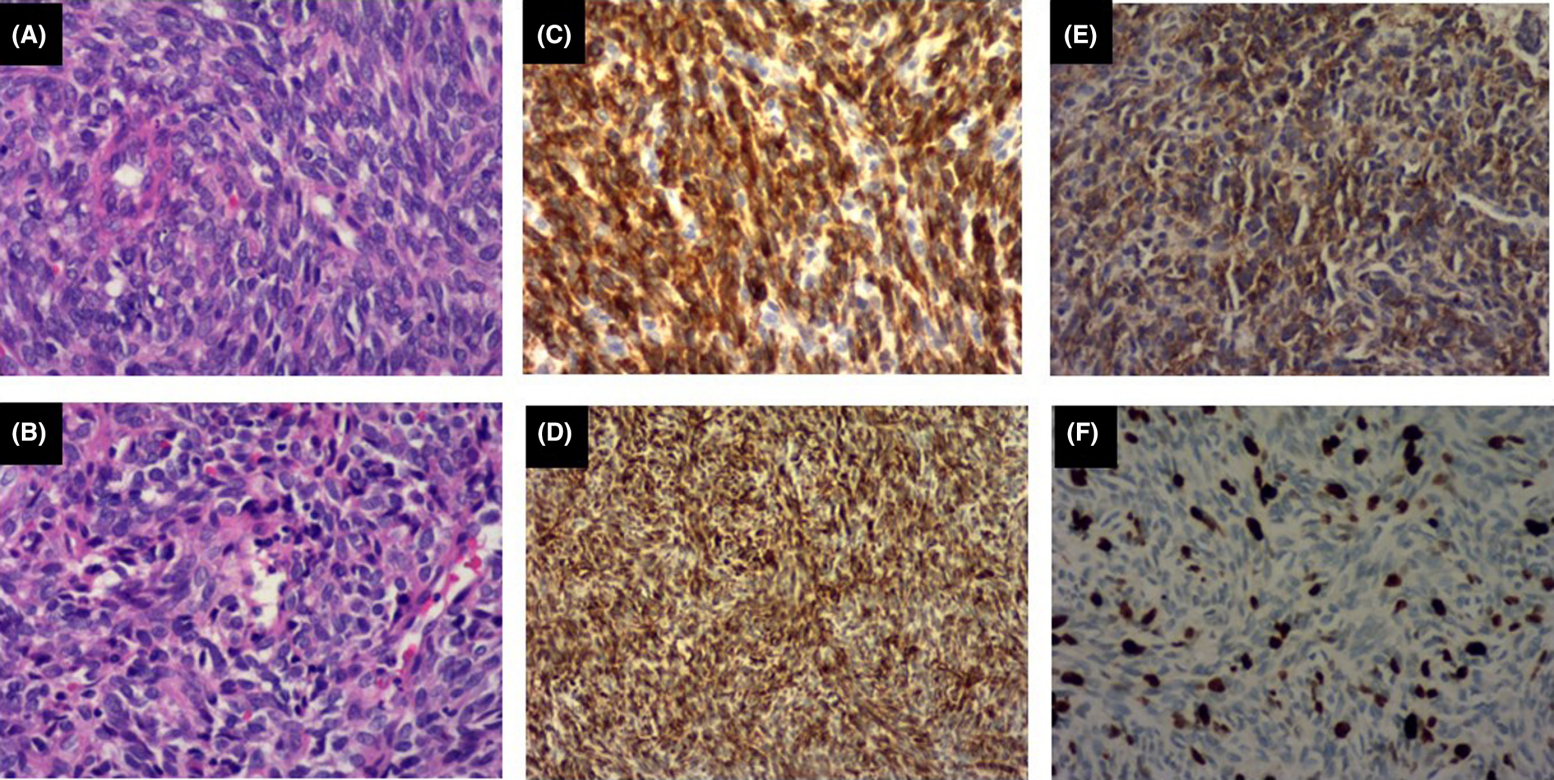
Pathology results. A–B: Routine hematoxylin and eosin staining. C–F: Immunohistochemical staining: Vimentin (+), Ki67(40%), Bc1‐2(+), and CD99(+). Original magnification 200×.

Postoperative pathology revealed a malignant spindle cell tumor. The histological morphology and immunolabeling results suggested a monophasic SS (2 cm × 1.8 cm × 1.2 cm). The tumor tissue was surrounded by hemorrhage, fibrosis, local alveolar epithelial hyperplasia, and bronchial rupture. The ends and broken ends of the blood vessels were not affected. Immunohistochemistry of an A4 slice revealed the following: TTF‐1 (+), NapsinA (+), P53 (<1%+), and Ki67 (5%+). Immunohistochemistry of an A1 slice revealed the following: CK (−), SMA (part +), EMA (partial +), P40 (one), CD117 (−), Vimentin (+), Ki67 (40% +), P53 (10% +), CD34 (vascular +), Bc1‐2 (+), CD99 (+), STAT6 (−), and Calponin (−) (Figure 3). The patient was successfully discharged after the surgery and refused further chemotherapy.

## 3 RESULTS

**FIGURE 4:**
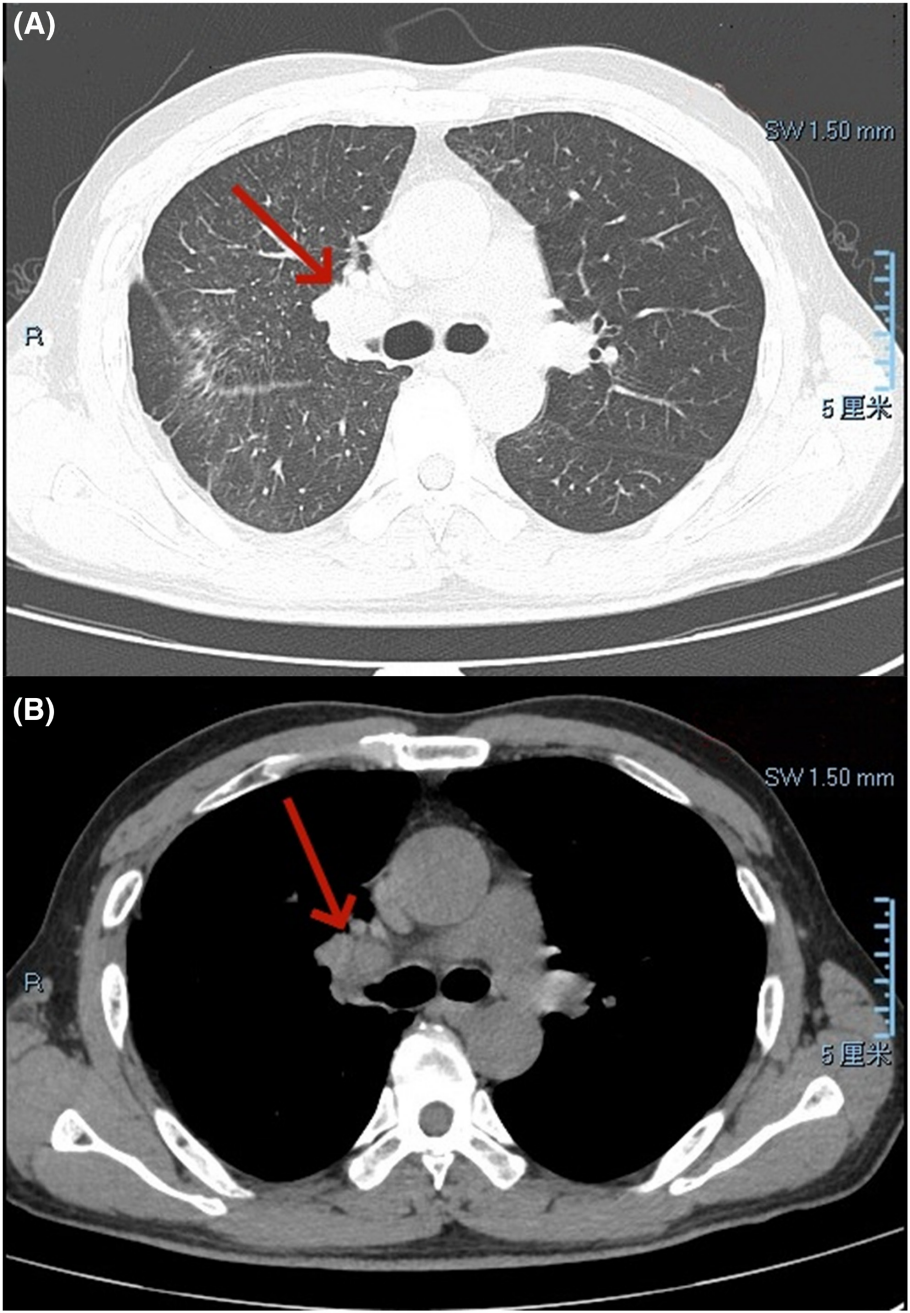
Chest computed tomography (CT) showed a mass occupying the right hilus, with unclear boundaries, size 4.6 × 3.6 cm. Tumor recurrence was diagnosed. (A) Represents the lung window manifestation on chest CT plain scan; and (B) Represents the mediastinal window manifestation.

A follow‐up chest CT scan at 7 months postoperatively revealed a mass‐like high‐density shadow with unclear boundaries in the right hilus, with a size of approximately 4.6 × 3.6 cm, and local calcifications (Figure 4). Multiple solid nodules were observed in the remaining two lungs. Tumor recurrence and metastasis were diagnosed, and the patient was not hospitalized for treatment.

## 4 DISCUSSION

Primary pulmonary synovial sarcoma (PPSS) has a low incidence; the most common symptoms are chest pain, cough, dyspnea, and hemoptysis. A study of 19 cases of PPSS found that pleural effusion was the least common manifestation.^4^ Our patient had a lung mass caused by a rare bloody pleural effusion and underwent surgery. He also exhibited symptoms of dyspnea and hemoptysis.

PPSS mostly occurs in patients aged 30–50 years old, with similar incidence rates between men and women.^5^ There are also reports that PPSS occurs in young and middle‐aged individuals who do not smoke,^6^ and in the upper and lower lobes of the lungs.^7^ Our patient's tumor was located in the middle lobe of the right lung near the hilum. Due to the low incidence of PPSS, there is a lack of characteristic imaging manifestations, which mainly include the following: a round solid mass with smooth edges, which may be accompanied by necrosis, liquefaction, and calcification, without cavities, burrs, and bronchial traction^8^; and manifests as nodular calcification, especially when present as a plaque or punctate calcification.^9^ However, there are few reports of intratumoral calcification in PPSS in the literature. The preoperative CT report of this case did not show intratumoral calcification; however, the follow‐up CT examination 7 months after the operation showed that there may have been tumor recurrence, and imaging showed intratumoral calcification. Although no pathological diagnosis was made, it could not be determined whether the tumor was of the same type as the surgically removed tumor. However, based on imaging findings, the tumor was hypothesized to be a recurrent PPSS.

Because monophasic SS has a uniform spindle cell pattern, PPSS is difficult to diagnose using imaging studies and is indistinguishable from other malignant spindle cell neoplasms. Presently, the diagnosis of PPSS mainly includes the following aspects. (1) Fine‐needle aspiration, which has been reported to be a reliable tool for the specific diagnosis of SS. When combined with the auxiliary molecule SS18 test, an accuracy of close to 100% was achieved.^10^ (2) Immunohistochemistry shows that monophasic subtypes can be mixed with other types of sarcomas; therefore, immunohistochemistry is crucial for differential diagnosis and can be used to confirm the diagnosis.^6^ (3) PPSS is usually positive for cytokeratin, EMA, BCL‐2, Vimentin, CD56 and CD99, TLE1, AE1\/AE3, CK7, and SMA,^6^, ^11^ and negative for S‐100 and Desmin. In the reported cases, both SMA and EMA were (partially +) and BCL‐2 was (+). (4) Cytogenetic testing and PPSS histological features are specific (X; 18) (p11.2; q11.2) translocations that produce the SS18‐SSX fusion gene, which has been used to diagnose this disease, which is the gold standard for histopathologically complex cancers.^7^ (5) In addition, there are also reports in the literature of fluorescent in situ hybridization detection using an SS dual‐color fracture separation probe used to diagnose SS with high sensitivity and specificity.^12^

Because of the low incidence of PPSS, its optimal treatment remains unknown. Current clinical treatments for PPSS include surgical resection, chemotherapy, radiotherapy, molecular‐targeted therapy, and immunotherapy. In recent years, the treatment for SS has significantly shifted toward radical resection combined with radiotherapy and/or chemotherapy. Regarding surgical treatment, because PPSS tumors are large and invasive, extensive surgical resection and surgical margins are crucial for preventing local recurrence. Normal tissue around the tumor is usually located 2 cm away.^13^ Surgical resection is the first‐line treatment for isolated PPSS, and lobectomy is the preferred surgical method.^7^ Furthermore, SS is a chemotherapy‐sensitive tumor. Adriamycin, alone or in combination with ifosfamide, is considered the standard chemotherapy regimen for SS and can achieve good local control. The overall response rate to combined chemotherapy was reported to be approximately 50%, which is higher than that of any single drug treatment.^12^ Regarding molecular‐targeted therapies and immunotherapies, pazopanib is an oral vascular endothelial growth factor receptor inhibitor that has been effectively used in patients with SS at any location.^14^ A clinical trial showed that pazopanib improved the prognosis of SS and extended the median progression‐free survival by 3 months (4.1 vs 1.0 months).^13^ Because most primary synovial sarcoma tumors also contain the SS18‐SSx fusion oncogene, several new therapeutic agents targeting this oncogene have been developed and are currently undergoing preliminary trials.

Unfortunately, this case refused further postoperative chemotherapy. The prognosis of PPSS is poor, and the 5‐year overall survival rate of SS in the lungs and bronchi is reported to be 41.80%, which is significantly lower than the average rate of SS (65.2%).^7^ There was also a reported difference in survival between surgical and nonsurgical patients, with a median overall survival ranging from 22 to 39.6 months in resected PPSS compared to 4.9 months in unresectable patients. Current studies have found that the main poor prognostic factors of PPSS are larger tumors, extensive tumor necrosis, a large number of mitoses (10\/10HPF), neurovascular invasion, lymph node invasion or distant metastasis, and SYT–SSX1 mutations. Our patient's tumor had a maximum diameter of 2.9 cm and no tumor necrosis, neurovascular invasion, lymph node invasion, or distant metastasis, suggesting a good prognosis. However, during chest CT reexamination 7 months after the operation, a mass was found in the right lung near the hilus, which was considered as recurrence of the tumor.

Reflecting on our entire treatment process, we only used warm saline to flush the remaining cavity at the end of the operation. If chemotherapy drugs were added to flush and kill locally shed cells, the recurrence rate could have been reduced. As a potential result, our patient was found to have a mass in the hilum again 7 months after the operation. Currently, there are few reports on the treatment of recurrent PPSS; however, there are reports on the treatment of recurrent SS. Surgical treatment and radical resection should be considered for surgically resectable lesions. For patients who are inoperable or have diffuse metastases, palliative treatment should be administered, including surgery, chemoradiotherapy, targeted therapy, interventional ablation, or hyperthermic infusion chemotherapy.^15^ However, the patient forfeited further treatment due to financial reasons.

## 5 CONCLUSION

Although the incidence rate of PPSS is low and rare in clinical practice, it has a high degree of malignancy, which should arouse our attention, especially for patients with chest pain, cough, dyspnea, and hemoptysis. Patients diagnosed with PPSS should actively receive further treatment, which can help prolong their survival.

## AUTHOR CONTRIBUTIONS

**Tengcheng Yin:** Conceptualization; data curation; writing – original draft. **Bing Liu:** Writing – review and editing. **Jinru Xue:** Writing – review and editing. **Xiyu Liu:** Writing – review and editing. **Shengtao Shang:** Writing – review and editing. **Yan Wang:** Funding acquisition; resources; writing – review and editing.

## CONFLICT OF INTEREST STATEMENT

The authors declare no conflicts of interest.

## ETHICS STATEMENT

This case report does not involve ethical issues. Informed consent was submitted by the subject.

## CONSENT

Written informed consent was obtained from the patient to publish this report in accordance with the journal's patient consent policy.

## Data Availability

The corresponding author's data supporting this study's findings are available upon reasonable request.
